# Ablation of Fam20c causes amelogenesis imperfecta via inhibiting Smad dependent BMP signaling pathway

**DOI:** 10.1186/s13062-020-00270-7

**Published:** 2020-10-07

**Authors:** Jing Liu, Wuliji Saiyin, Xiaohua Xie, Limin Mao, Lili Li

**Affiliations:** 1 Department of Stomatology, the 1st Affiliated Hospital of Harbin Medical University, 23 Youzheng Road, Nangang, Harbin, 150001 Heilongjiang China; 2 Institute of Hard Tissue Development and Regeneration, the 2nd Affiliated Hospital of Harbin Medical University, Harbin, 150086 Heilongjiang China; 3 Longjiang scholar laboratory, the 1st Affiliated Hospital of Harbin Medical University, 23 Youzheng Road, Nangang, Harbin, 150001 Heilongjiang China

**Keywords:** FAM20C, Amelogenesis Imperfecta, BMP signaling pathway, Ameloblast differentiation

## Abstract

**Background:**

Amelogenesis imperfecta (AI) is a type of hereditary diseases that manifest defects in the formation or mineralization of enamel. Recently, it is reported that inactivation of FAM20C, a well-known Golgi casein kinase, caused AI. However, the mechanism of it is still unknown. The aim of this study was to explore the molecular mechanism of AI, which caused by ablation of FAM20C.

**Results:**

In the *Sox2-Cre;Fam20C*^*fl/fl*^ (cKO) mouse, we found abnormal differentiation of ameloblasts, improper formation and mineralization of enamel, and downregulation of both mRNA and protein level of enamel matrix proteins, including amelogenin (AMEL), ameloblastin (AMBN) and enamelin (ENAM). The levels of BMP2, BMP4 and BMP7, the ligands of BMP signaling pathway, and phosphorylation of Smad1/5/8, the key regulators of BMP signaling pathway, were all decreased in the enamel matrix and the ameloblast of the cKO mice, respectively. The expression of cyclin-dependent kinase inhibitor (P21), muscle segment homeobox genes 2 (Msx2), which are the target genes of the BMP signaling pathway, and laminin 3, the downstream factor of Msx2, were all significantly decreased in the ameloblasts of the cKO mice compared to the control mice.

**Conclusion:**

the results of our study suggest that ablation of FAM20C leads to AI through inhibiting the Smad dependent BMP signaling pathway in the process of amelogenesis.

## Background

Amelogenesis imperfecta (AI) is a type of hereditary diseases that manifest defects in the formation or mineralization of enamel [1]. AI exhibit abnormally thin, soft, fragile, pitted and discolored enamel phenotype. Patients with AI often have some problems such as early tooth loss, eating difficulties, and pain. Therefore, understanding the biological mechanism of AI is essential for providing effective and potential clinical treatment for AI.

There are many reasons for AI. During the secretory stage, ameloblasts secreted abundant enamel matrix proteins (EMPs), such as amelogenin (AMEL), ameloblastin (AMBN) and enamelin (ENAM), and numerous cell adhesion molecules and it is reported that mutations of these EMPs will cause Hypoplastic AI in human [2–7]. Hypomineralized AI is caused by maturation stage failure, giving rise to enamel that is of full thickness but is weak and fails prematurely [8]. During maturation stage, ameloblasts secrete two kinds of matrix proteases which are Matrix metallopeptidases 20 (MMP20) and Kallikrein-4 (KLK4). In human/mouse the mutations of either *Mmp20* or *Klk4* gene will lead to AI [9, 10]. Beside the EMPs and the enamel matrix proteases, dysfunction of some growth factors, which regulating signaling pathways that enrolled in amelogenesis, could also result in AI.

BMP signaling pathway is one of the important pathways that regulating amelogenesis. BMP2, BMP4 and BMP7, which are the main ligands of BMP signaling pathway, were reported to be expressed in the preameloblasts and ameloblasts and closely related to the formation of enamel [11]. The abrogation of *Bmp2* from differentiating odontoblasts of mice resulted in hypoplastic AI with a reduced expression of AMEL and AMBN and strong inhibition of the Smad dependent BMP signaling pathway in ameloblasts [12]. The deletion of *Bmp4* driven by *3.6 kb Col I* promoter also caused severe AI phenotype, the findings of which were similar to that of the deletion of *Bmp2* in the differentiating odontoblasts of mice [13]. However, *Bmp7*-deficient mice did not reveal obvious changes in enamel, possibly because of the functional redundancy with other BMP members [14]. Nevertheless, the exact roles of BMP2, BMP4 and BMP7 in regulating the formation of enamel are still unknown and need to be further explored.

FAM20C, a member of the family with sequence similarity 20 (FAM20), is a Golgi casein kinase that can phosphorylate over 1 hundred secreted proteins within Ser-x-Glu / phospho-Ser (SxE/pS) motifs [15]. Recently, it is reported that FAM20C is highly expressed in enamel and play vital role in amelogenesis. Xiaofang Wang et al. found that ubiquitously deletion of *Fam20C* cause severe AI like phenotype, indicating that FAM20C is enrolled in the process of amelogenesis and also revealed that the phosphorylation defects of EMPs caused by ablation of FAM20C may be the main reason for this. However, the exact molecular mechanism, for which ablation of FAM20C causes AI, is still not clear and need to be further studied.

In this study, we use the *Sox2-Cre;Fam20C*^*fl/fl*^ mouse to ubiquitously delete the expression of *Fam20C* in enamel to construct a model of AI. In this AI mouse model, we analyzed the effects of FAM20C ablation in amelogenesis and assessed the levels molecules associated with the activation of Smad dependent BMP signaling pathway in the ameloblast of *Sox2-Cre;Fam20C*^*fl/fl*^ mouse. The cKO mouse exhibited severe differentiation defect of ameloblast with downregulation of the gene expression of enamel matrix proteins encoding AMEL, AMBN and ENAM, along with altered levels of molecules associated with the activation of Smad dependent BMP signaling pathway. The findings in this investigation indicate that ablation of FAM20C resulted in the differentiation defect of ameloblast, a major contributor to AI, may be related to the inhibition of the Smad dependent BMP signaling pathways associated with the loss of FAM20C function in the ameloblasts.

## Results

### Validation of Fam20C ablation in the ameloblasts of cKO mice

**Fig. 1:**
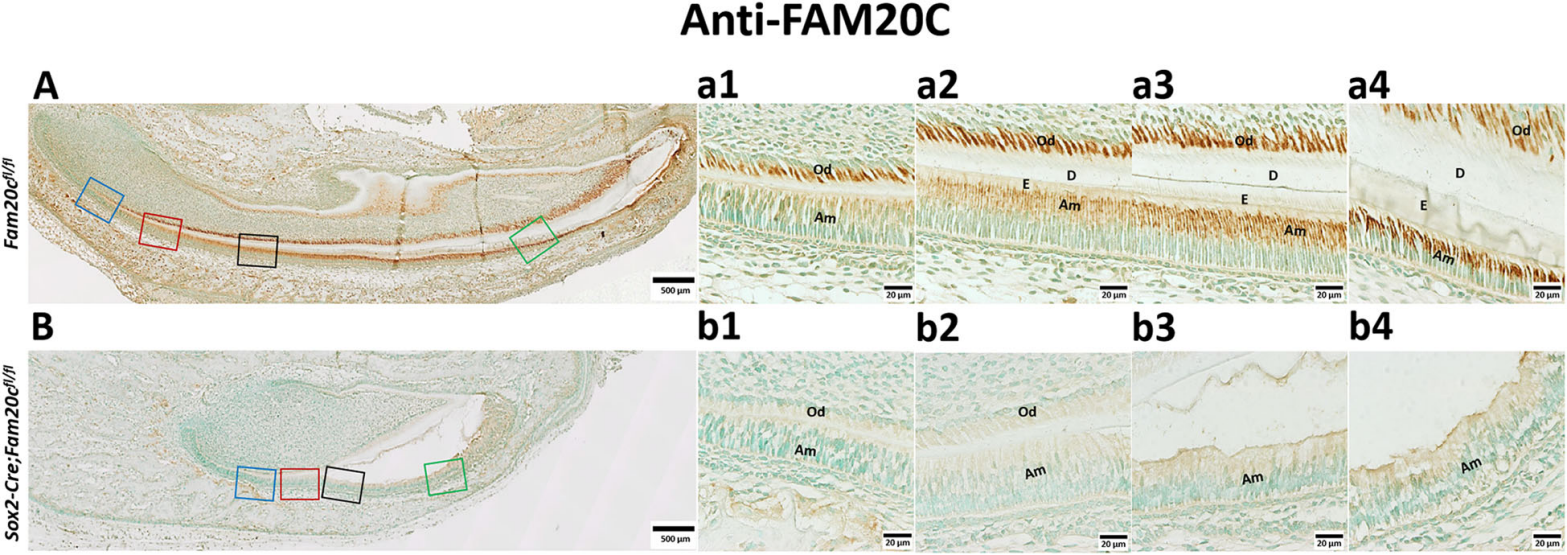
Reduced expressions of FAM20C in the *Sox2-Cre;Fam20C*^*fl/fl*^ mice. **A** and **B**, IHC analyses (signal in brown) of FAM20C on the sagittal sections of the mandibular incisors of 7-day-old *Fam20c*^*fl/fl*^ (**A**) and *Sox2-Cre;Fam20c*^*fl/fl*^ mice (**B**). a1, a2, a3, a4, b1, b2, b3 and b4 are the higher magnification views of the preameloblasts in blue boxes, the presecretory ameloblasts in red boxes, the secretory ameloblasts in purple boxes, the maturation ameloblasts in green boxes in **A** and **B**, respectively. FAM20C was absent in the incisors of *Sox2-Cre;Fam20c*^*fl/fl*^ mouse. Scale bars: 500 μm in **A** and **B**; 20 μm in a1, a2, a3, a4, b1, b2, b3 and b4. (E: Enamel; D: dentin; Am: Ameloblast; Od: Odontoblast)

By crossbreeding the *Fam20C*^*flox/flox*^ mice with the transgenic mice expressing Cre-recombinase driven by the *Sox2* promoter, we generated the *Sox2-Cre;Fam20C*^*flox/flox*^ mice (referred to as “cKO mice” in this report). We characterized cKO mice, in comparison with the *Fam20C*^*flox/flox*^ littermates that served as normal controls (Ctrl). As Fig. 1B shows, anti-FAM20C IHC analyses revealed that FAM20C was almost absent in all stages of ameloblasts, including preameloblast, presecretory ameloblasts, secretory ameloblasts and maturation ameloblasts, in cKO mice. However, in the normal control mice FAM20C was evenly distributed in the ameloblasts, and the level of FAM20C increases as the maturation stage of ameloblasts improves (Fig. 1A).

### Ablation of Fam20C causes AI

**Fig. 2:**
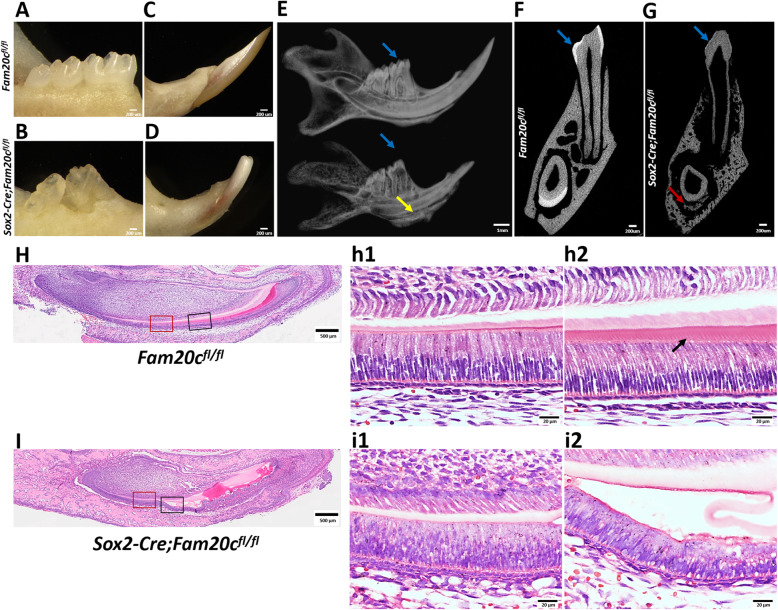
Histological analysis of the enamel defects in the *Sox2-Cre;Fam20C*^*fl/fl*^ mice. **A** and **B**, photographs of the mandibular molars of 7-week-old *Fam20c*^*fl/fl*^ and *Sox2-Cre;Fam20c*^*fl/fl*^ mice. **C** and **D**, photographs of the mandibular incisors of 7-week-old *Fam20c*^*fl/fl*^ and *Sox2-Cre;Fam20c*^*fl/fl*^ mice. **E**, plain x-ray images of the mandibles of 7-week-old *Fam20c*^*fl/fl*^ (the picture above)and *Sox2-Cre;Fam20c*^*fl/fl*^ (the picture below)mice. **F** and **G** are the reconstructed trans-axial μCT images for molar and incisor enamel. The blue arrows in **E**, **F** and **G** indicate the enamel of first molars. The yellow arrow in **E** shows the ectopic calcifications in the *Sox2-Cre;Fam20c*^*fl/fl*^ mice. The red arrow in **G** shows the malformed enamel in incisors of the *Sox2-Cre;Fam20c*^*fl/fl*^ mice. **H** and **I**, **H**&**E** staining of the sagittal sections of the mandibular incisors of 3-day-old *Fam20c*^*fl/fl*^ and *Sox2-Cre;Fam20c*^*fl/fl*^ mice. h1, h2, i1 and i2 are the higher magnification views of the presecretory ameloblasts in red boxes and the secretory ameloblasts in black boxes in **H** and **I**, respectively. In the *Fam20c*^*fl/fl*^ mice, Tomes’ processes (pointed by black arrow in h2) were clearly visible, whereas they were lost in the *Sox2-Cre;Fam20c*^*fl/fl*^ mice (i2). Scale bars: 200 μm in **A**-**D**, **F** and **G**; 1 mm in **E**; 500 μm in **H** and **I**; 20 μm in h1, h2, i1 and i2

The cKO mice showed apparent enamel defects compared to the normal controls. The mandible first molars of 7-week-old cKO mice displayed yellowish and rough surface, likely resulting from loss of enamel and exposure of the underlying dentin (Fig. 2A, B). The incisors of cKO mice exhibited a shorter and chalky white appearance (Fig. 2C, D). Plain x-ray radiography showed that the mandibular molars and incisors had no sharp cups, became blunt and shorter in the cKO mice. In addition to this, we also noticed some lower density sediment-like tissues located between the alveolar bone and the labial side of incisors in the cKO mice (Fig. 2E). μCT analysis showed that the incisors had a distinct layer of enamel formed on their labial side in the control mice whereas a layer of ectopic calcifications, in place of enamel, was found on the labial side of the incisors in the cKO mice. Moreover, there was no enamel formed on the mandibular first molars of the cKO mice (Fig. 2G).

H&E staining showed that the presecretory ameloblasts of control mice exhibited a normal polarity with their apical ends against the enamel matrix (Fig. 2H, h1), whereas the ameloblasts in the cKO mice showed poor polarity and disorganization (Fig. 2I, i1). The secretory ameloblasts of control mice showed a high columnar shape with Tome’s process on their apical ends (Fig. 2H, h2). However, the secretory ameloblasts of the cKO mice became dramatically shorter, lacked Tome’s process, and detacted from the enamel matrix (Fig. 2I, i2).

**Fig. 3:**
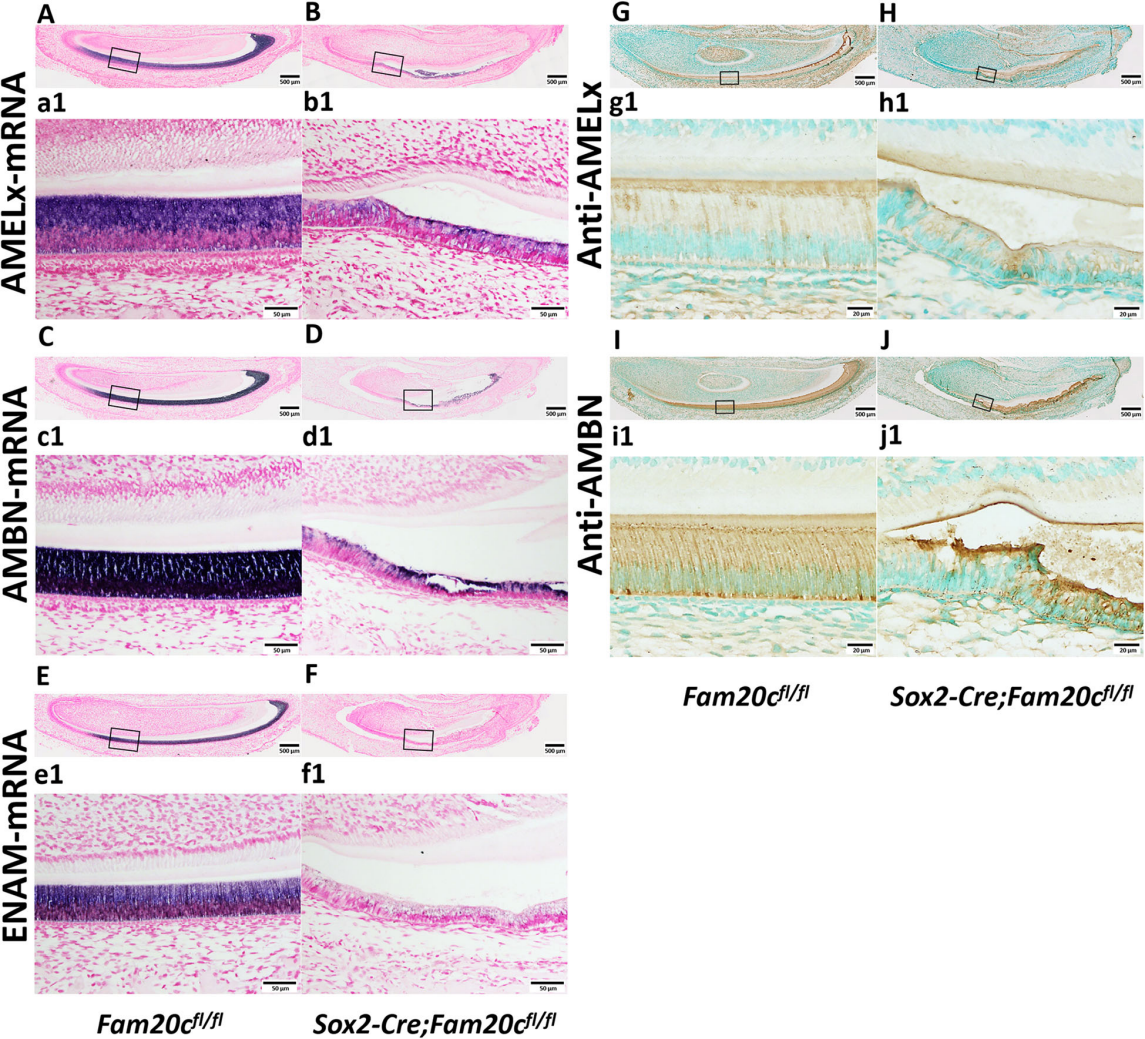
Reduced expressions of the secretory ameloblast markers in the *Sox2-Cre;Fam20C*^*fl/fl*^ mice. **A**-**F**, ISH analyses (signal in purple) of AMEL (**A** and **B**), AMBN (**C** and **D**) and ENAM (**E** and **F**) on the sagittal sections of the mandibular incisors of 3-day-old *Fam20c*^*fl/fl*^ (**A**, **C** and **E**) and *Sox2-Cre;Fam20c*^*fl/fl*^ (**B**, **D** and **F**) mice. a1, b1, c1, d1, e1 and f1 are the higher magnification views of the secretory ameloblasts in black boxes in **A**, **B**, **C**, **D**, **E** and **F**, respectively. The expressions of AMEL, AMBN and ENAM were sharply decreased in the incisors of *Sox2-Cre;Fam20c*^*fl/fl*^ mouse compared to the *Fam20c*^*fl/fl*^ mouse. IHC analyses of AMEL (G and H) and AMBN (**I** and **J**) on the sagittal sections of the mandibular incisors of 3-day-old *Fam20c*^*fl/fl*^ (**G** and **I**) and *Sox2-Cre;Fam20c*^*fl/fl*^ (**H** and **J**) mice. g1, h1, i1 and j1 are the higher magnification views of the secretory ameloblasts in black boxes in **G**, **H**, **I** and **J**, respectively. Both the protein level of AMEL and AMBN were markedly reduced in the incisors of *Sox2-Cre;Fam20c*^*fl/fl*^ mouse compared to the *Fam20c*^*fl/fl*^ mouse. Scale bars: 500 μm in **A** - **J**; 50 μm in a1, b1, c1, d1, e1, f1; 20 μm in g1, h1, i1, j1

To further analyze the enamel defects caused by the ablation of FAM20C, we detected the level of AMEL, AMBN and ENAM, all of which are the well-known ameloblast markers, by ISH and/or IHC. The results of ISH showed that the expression of AMEL, AMBN and ENAM were remarkably decreased in the ameloblasts of cKO mice, compared to the control mice (Fig. 3A, B, C, D, E, F). IHC detection also demonstrated that the protein level of AMEL and AMBN dramatically decreased in the cKO mice than in the control mice (Fig. 3G, H, I, J). Taken together, these results demonstrated that deletion of FAM20C results in serious amelogenesis imperfecta.

### Ablation of FAM20C inhibits the activation of BMP signaling pathway in ameloblasts

**Fig. 4:**
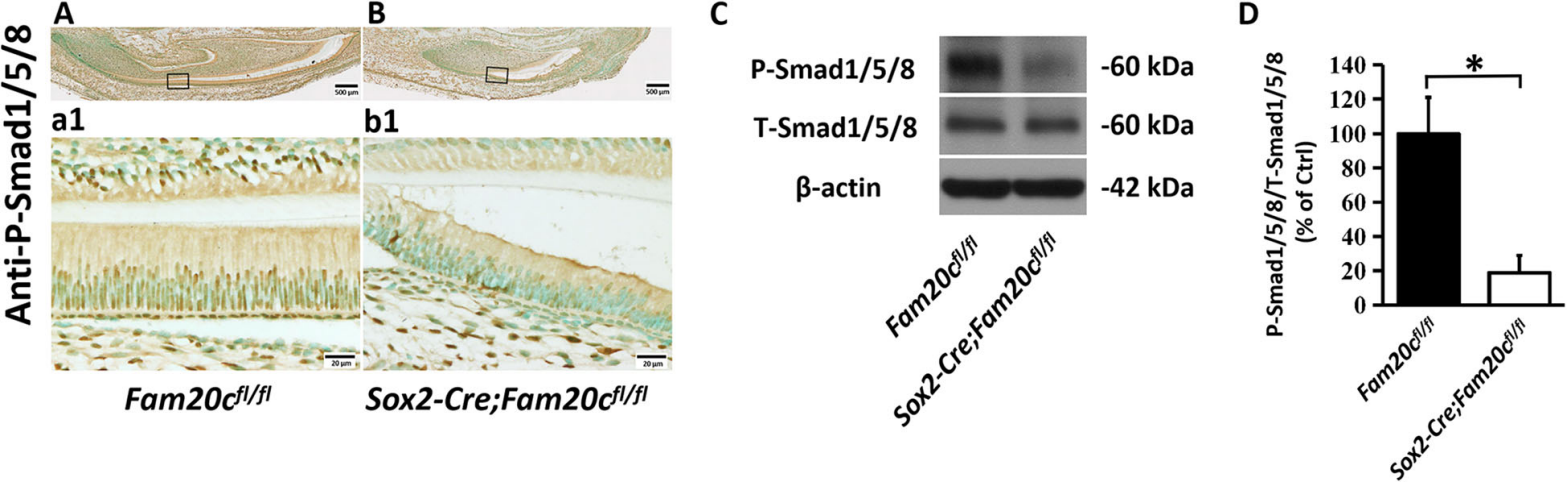
Inhibition of BMP signaling pathway in the *Sox2-Cre;Fam20c*^*fl/fl*^ mice. **A** and **B**, IHC analysis of P-Smad1/5/8 on the sagittal sections of the mandibular incisors of 3-day-old *Fam20c*^*fl/fl*^ and *Sox2-Cre;Fam20c*^*fl/fl*^ mice. a1 and b1 are the higher magnification views of the secretory ameloblasts in black boxes in **A** and **B**, respectively. The level of P-Smad1/5/8 was markedly reduced in the *Sox2-Cre;Fam20c*^*fl/fl*^ mice than in the *Fam20c*^*fl/fl*^ mice. **C**, Western-immunoblotting analyses of P-Smad1/5/8 and T-Smad1/5/8 of the enamel organ cells of 3-day-old *Fam20c*^*fl/fl*^ and *Sox2-Cre;Fam20c*^*fl/fl*^ mouse incisors. **D**, Relative protein levels of P-Smad1/5/8 to total P-Smad1/5/8 determined by Western immunoblotting (*n* = 3; * = *p* < 0.05). The protein levels of P-Smad1/5/8 were significantly decreased in the enamel organ cells of *Sox2-Cre;Fam20c*^*fl/fl*^ mice compared to the *Fam20c*^*fl/fl*^ mice, while the protein levels of T-Smad1/5/8 were not changed. Scale bars: 500 μm in **A** and **B**, respectively and 20 μm in a1 and b1, respectively

BMP signaling pathway is known to be essential for the tooth development [16]. The activation of BMP signaling pathway depends on the phosphorylation of Smad 1, Smad 5 and Smad 8. In this study, we examined the levels of phospho-Smad 1/5/8 in the ameloblasts of the control and cKO mice by immunohistochemistry and Western immunoblotting analyses. As shown in Fig. 4A and B, the numbers of cells positive for phospho-Smad 1/5/8 were much fewer in the ameloblasts of cKO mice than in the control mice. Western immunoblotting analyses of total proteins extracted from the layer of ameloblasts revealed that the levels of phospho-Smad 1/5/8 were significantly lowered in the cKO mice (Fig. 4C). However, the total protein levels of Smad1/5/8 were not changed. The ratios of phospho-Smad 1/5/8 to total-Smad 1/5/8 were significantly decreased in the cKO mice (Fig. 4D). These results indicate that in the ameloblasts of cKO mice the activation of Smad-dependent BMP signaling pathway may be inhibited.

**Fig. 5:**
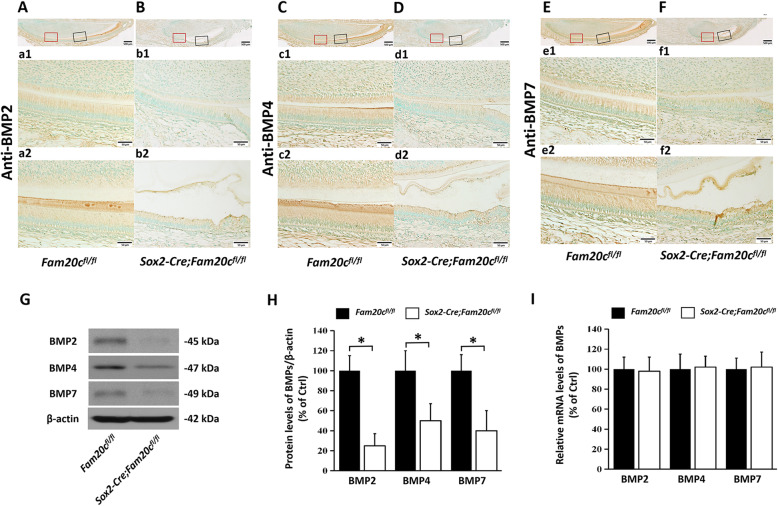
BMPs was decreased dramatically in the secretory ameloblasts of the *Sox2-Cre;Fam20c*^*fl/fl*^ mice. **A** and **B**, IHC analyses (signal in brown) of BMP2 on the sagittal sections of the mandibular incisors of 3-day-old *Fam20c*^*fl/fl*^ (**A**) and *Sox2-Cre;Fam20c*^*fl/fl*^ mouse (**B**) a1 and b1 are the higher magnification views of the presecretory ameloblasts in red boxes, a2 and b2 are the secretory ameloblasts in black boxes in **A** and **B**, respectively. BMP2 was significantly reduced in the incisors of *Sox2-Cre;Fam20c*^*fl/fl*^ mouse compared to the *Fam20c*^*fl/fl*^ mouse. **C** and **D**, IHC analyses of BMP4 on the sagittal sections of the mandibular incisors of 3-day-old *Fam20c*^*fl/fl*^ (**C**) and *Sox2-Cre;Fam20c*^*fl/fl*^ mice (**D**). c1 and d1 are the higher magnification views of the presecretory ameloblasts in red boxes, c2 and d2 are the secretory ameloblasts in black boxes in **C** and **D**, respectively. BMP4 was significantly reduced in the incisors of *Sox2-Cre;Fam20c*^*fl/fl*^ mouse compared to the *Fam20c*^*fl/fl*^ mouse. **E** and **F**, IHC analyses of BMP7 on the sagittal sections of the mandibular incisors of 3-day-old *Fam20c*^*fl/fl*^ (**E**) and *Sox2-Cre;Fam20c*^*fl/fl*^ mice (**F**). e1 and f1 are the higher magnification views of the presecretory ameloblasts in red boxes, e2 and f2 are the secretory ameloblasts in black boxes in **E** and **F**, respectively. BMP7 was also reduced significantly in the *Sox2-Cre;Fam20c*^*fl/fl*^ mouse incisors, compared to the *Fam20c*^*fl/fl*^ mouse incisors. **G**, Western-immunoblotting analyses of BMP2, BMP4 and BMP7 of the enamel organ cells of 3-day-old *Fam20c*^*fl/fl*^ and *Sox2-Cre;Fam20c*^*fl/fl*^ mouse incisors. **H**, Relative protein levels of BMP2, BMP4 and BMP7 determined by Western immunoblotting (*n* = 3; * = *p* < 0.05). The protein levels of BMP2, BMP4 and BMP7 were significantly decreased in the enamel organ cells of *Sox2-Cre;Fam20c*^*fl/fl*^ mice compared to the *Fam20c*^*fl/fl*^ mice. **I**, Real-time PCR analyses of the mRNA levels of the *Bmp2*, *Bmp4* and *Bmp7* in the enamel organ cells of 3-day-old *Fam20c*^*fl/fl*^ and *Sox2-Cre;Fam20c*^*fl/fl*^ mouse incisors. The mRNA levels of *Bmp2*, *Bmp4* and *Bmp7* were not changed in the enamel organ cells of *Sox2-Cre;Fam20c*^*fl/fl*^ mice compared to the *Fam20c*^*fl/fl*^ mice. Scale bars: 500 μm in **A** - **F**; 50 μm in a1- f1; 50 μm in a2 - f2

To further investigate the activation of BMP signaling pathway in ameloblasts of cKO mice, we analyzed the protein levels of BMP2, BMP4 and BMP7 in the enamel matrix. BMP2, BMP4 and BMP7 are the important ligands, which are essential for amelogenesis, of BMP signaling pathway. IHC analyses showed that the protein levels of BMP2, BMP4 and BMP7 were lower in the enamel matrix of cKO mice than in the normal control mice (Fig. 5A, B, C, D, E, F). Western immunoblotting analyses of proteins extracted from the enamel confirmed that the enamel matrix of cKO mice had reduced level of BMP2, BMP4 and BMP7 (Fig. 5G, H). However, the gene expressions of *Bmp2*, *Bmp4* and *Bmp7* were not changed (Fig. 5I).

**Fig. 6:**
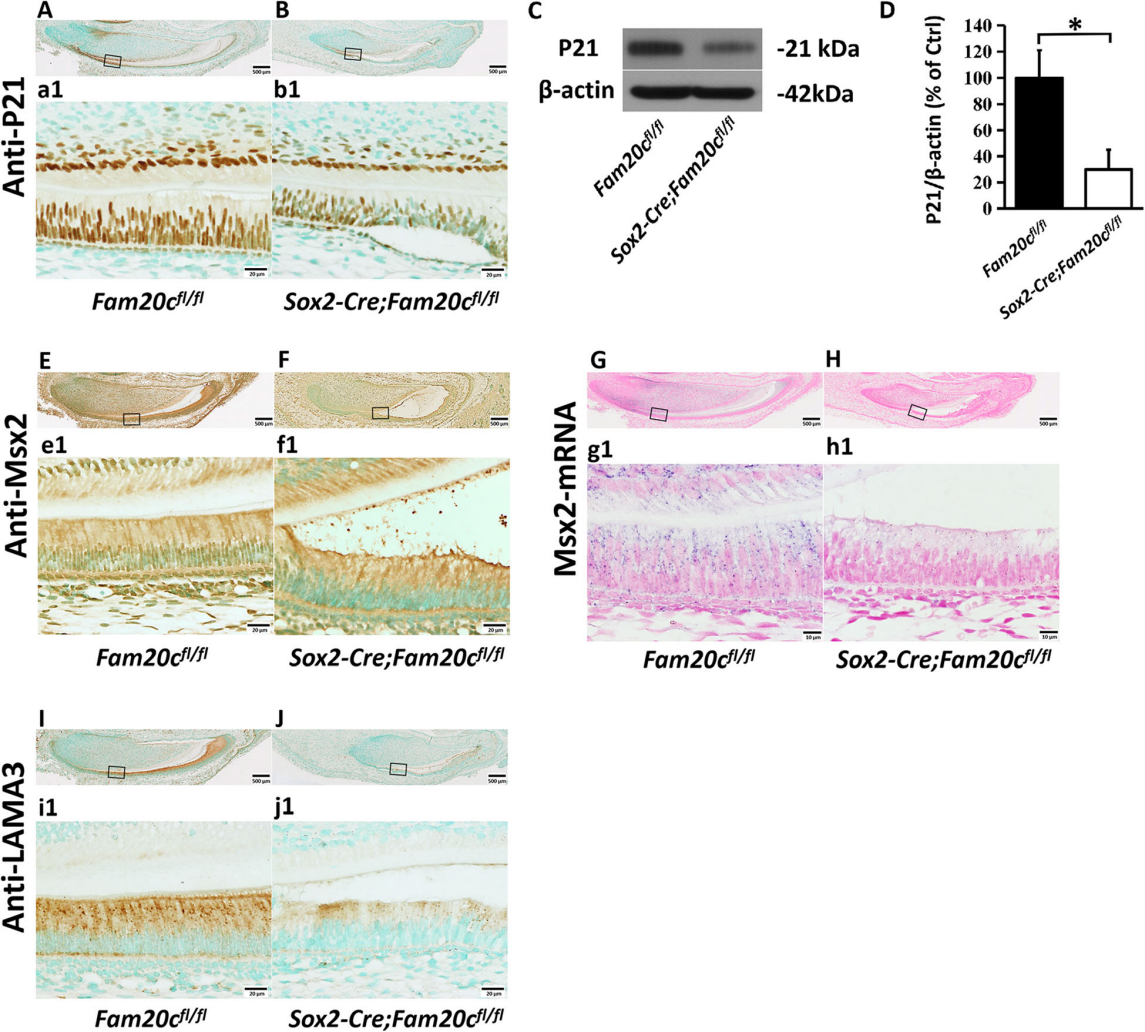
Detection of the BMP signaling pathway target genes in the *Sox2-Cre;Fam20c*^*fl/fl*^ mice. **A** and **B**, IHC analyses (signal in brown) of P21 on the sagittal sections of the mandibular incisors of 3-day-old *Fam20c*^*fl/fl*^ (**A**) and *Sox2-Cre;Fam20c*^*fl/fl*^ mice (**B**). a1 and b1 are the higher magnification views of the presecretory ameloblasts in black boxes in **A** and **B**, respectively. P21 was reduced significantly in the incisors of *Sox2-Cre;Fam20c*^*fl/fl*^ mouse compared to the *Fam20c*^*fl/fl*^ mouse. **C**, Western-immunoblotting analyses of P21 in the enamel organ cells of 3-day-old *Fam20c*^*fl/fl*^ and *Sox2-Cre;Fam20c*^*fl/fl*^ mouse incisors. **D**, Relative protein levels of P21 determined by Western immunoblotting (*n* = 3; * = *p* < 0.05). The protein level of P21 was significantly decreased in the enamel organ cells of the *Sox2-Cre;Fam20c*^*fl/fl*^ mice compared to the *Fam20c*^*fl/fl*^ mice. **E** and **F**, IHC analysis of MSX2 on the sagittal sections of the mandibular incisors of 3-day-old *Fam20c*^*fl/fl*^ and *Sox2-Cre;Fam20c*^*fl/fl*^ mice. e1 and f1 are the higher magnification views of the secretory ameloblasts in black boxes in **E** and **F**, respectively. The *Sox2-Cre;Fam20c*^*fl/fl*^ mice had reduced nuclear localization of MSX2 in ameloblasts compared to that of the *Fam20c*^*fl/fl*^ mice. **G** and **H**, ISH analyses (signal in purple) of Msx2 mRNA on the sagittal sections of the mandibular incisors of newborn *Fam20c*^*fl/fl*^ and *Sox2-Cre;Fam20c*^*fl/fl*^ mice. g1 and h1 are the higher magnification views of the secretory ameloblasts in black boxes in **G** and **H**, respectively. The mRNA level of Msx2 was reduced in the ameloblasts of *Sox2-Cre;Fam20c*^*fl/fl*^ mice, compared to the control mice. **I** and **J**, IHC analysis of LAMA3 on the sagittal sections of the mandibular incisors of 3-day-old *Fam20c*^*fl/fl*^ and *Sox2-Cre;Fam20c*^*fl/fl*^ mice. i1 and j1 are the higher magnification views of the secretory ameloblasts in black boxes in **I** and **J**, respectively. The level of LAMA3 was markedly reduced in the *Sox2-Cre;Fam20c*^*fl/fl*^ mice compared to the control mice. Scale bars: 500 μm in **A**, **B**, **E**, **F**, **G**, **H**, **I** and **J**; 20 μm in a1, b1, e1, f1, i1 and j1; 10 μm in g1 and h1

P21, which enrolled in the development of ameloblast, is a target gene of BMP signaling pathway. Immunohistochemistry showed that the P21 positive cells were dramatically reduced in the ameloblasts of the 3-day-old cKO mice compared to the control mice (Fig. 6A, B). Western immunoblotting analysis of P21 obtained similar results (Fig. 6C, D). Msx2 is another target gene of BMP signaling pathway. It is reported that Msx2 is essential for the development of ameloblast. The results of immunohistochemistry indicate that the protein level of Msx2 in the ameloblasts of cKO mice was apparently decreased (Fig. 6E, F). Besides, we detected the mRNA level of Msx2 by ISH and noticed that the mRNA level of Msx2 was also dramatically downregulated (Fig. 6G, H). *Lama3* is a subunit of laminin5 and a downstream gene of *Msx2*. Lama3 is a kind of secreted protein, which is vital for the binding of ameloblasts with enamel matrix. In this study, we found that the protein level of Lama3 was markedly reduced in enamel matrix of cKO mice (Fig. 6I, J). Collectively, these results indicated that the ablation of FAM20C inhibited the proper activation of BMP signaling pathway in ameloblast.

## Discussion

In this study, we created a FAM20C conditional knockout mouse model (*Sox2-Cre;Fam20C*^*fl/fl*^) in which FAM20C was ablated in both ameloblast and odontoblasts. We found that the *Sox2-Cre;Fam20C*^*fl/fl*^ mice developed Amelogenesis Imperfecta phenotype, which is consistent with the results of the previous studies. Using the continuously growing mandibular incisors from these *Sox2-Cre;Fam20C*^*fl/fl*^ mice, we further studied the enamel phenotype caused by ablation of FAM20C. We found that the secretory ameloblasts were shorter, and subsequently lost their polarity, became disorganized and formed numerous spherical extracellular matrixes in place of normal enamel. Besides, the cKO mice showed severe preameloblast and ameloblast differentiation defects and down-regulated gene expression of enamel matrix proteins encoding AMEL, AMBN and ENAM. These results indicate that FAM20C not only phosphorylate the EMPs, which is proved by previous studies, but also promotes the differentiation and maturation of ameloblast. Therefore, we consider that beside causing the phosporylation defects of EMPs in AI, ablation of FAM20C may also affects the proper activation of some signaling pathways, which enrolled in the differentiation and maturation of ameloblast.

BMP signaling pathway, especially the Smad dependent BMP signaling pathway, plays vital role in the differentiation and maturation of ameloblast [11, 17]. To study whether the BMP signaling pathway was affected in the process of amelogenesis in the cKO mice, we studied the protein level of BMP ligands - BMP2, BMP4 and BMP7 and found that all of these ligands were dramatically reduced in the enamel of cKO mice with the gene expressions of them were not changed. Furthermore, we also noticed that the phosphorylation of Smad1/5/8, which are the key regulators of Smad dependent BMP signaling pathway, was significantly decreased in the ameloblast of cKO mice than in the control mice. These results indicate that the Smad dependent BMP signaling pathway was inhibited in the ameloblast of cKO mice. Recently, BMP4 has been experimentally confirmed to be a FAM20C-dependent phosphorite [15]. The other BMPs, like BMP2 and BMP7, have a similarity in the protein structures of BMP4 and may also be phosphorylated by FAM20C. Therefore, we believe that the phosphorylation of BMPs by FAM20C is essential to the normal biological function of BMPs, including the function of BMPs as ligands to interact with their receptors to activate BMP signaling pathway. In this study, the ablation of FAM20C may leads to the phosphorylation defects of BMPs and downregulate the biological functions of BMPs and then suppress the activation of Smad dependent BMP signaling pathway.

To further study the inhibition of Smad dependent BMP signaling pathway in the ameloblast of cKO mice, we studied the expression of P21 and Msx2, two target genes of the Smad dependent BMP signaling pathway, in the ameloblast of cKO mice. P21 is an transcription factor, which is important for the withdrawal of preameloblasts from cell cycle and entering cell differentiation [18]. Msx2 is also a transcription factor that play vital role in differentiation of ameloblast [19]. Studies showed that Msx2 controlled the terminal differentiation of ameloblast by up-regulating the expression of Lama3, which had a closely association with cell adhesion [20]. In this study, we noticed that the gene expression and the protein levels of P21 and Msx2, were all reduced in the ameloblast of cKO mice. Furthermore, we also found that the protein level of Lama3 was significantly reduced in the cKO mice than in the control mice, likely due to the marked down-regulation of Msx2 protein level. Taken together, these results further confirmed the inhibition of the Smad dependent BMP signaling pathway in the ameloblast of the cKO mice, which is the possible reason for the differentiation and maturation defect of the ameloblast in cKO mice.

## Conclusion

To be concluded, our study showed that the inactivation of FAM20C suppress the proper activation of the Smad dependent BMP signaling pathway in ameloblast, which in turn inhibit the normal differentiation of the ameloblast and then leads to AI.

## Methods

### Generation of *Sox2-Cre; Fam20c*^*fl/fl*^ mice

The *Sox2-Cre;Fam20C*^*fl/fl*^ (cKO) mice were generated by breeding *Fam20C*^*fl/fl*^ with *Sox2-Cre* transgenic mice (the Jackson Laboratory). The *Fam20C*^*fl/fl*^ (control) mice from the same litters were used as controls. Mouse genotyping was performed by PCR analyses of genomic DNA extracted from tail biopsies. All animal procedures were approved by the Institutional Animal Care and Use Committee of Harbin Medical University (Harbin, China; approved protocol nos. SYDW2018–046) and performed in accordance with the National Institutes of Health Guide for the Care and Use of Laboratory Animals.

### Plain X-ray radiography and micro-computed tomography (μCT)

The mandibles dissected from 7-week-old *Fam20C*^*fl/fl*^ and *Sox2-Cre;Fam20C*^*fl/fl*^ mice were analyzed with plain X-ray radiography (Faxitron Bioptics, Tucson, AZ, USA) and μCT (μCT35, Scanco Medical, Brüttisellen, Switzerland), as we previously described [21].

### Tissue processing and histological analysis

The mouse mandibles were processed for standard paraffin embedding, and 5-μm serial sections were cut and used for Hematoxylin and Eosin (H&E) staining, in situ hybridization (ISH) and immunohistochemistry (IHC).

ISH was performed to examine the expressions of AMEL, AMBN, ENAM and Msx2, as described previously [22]. Briefly, RNA probes were labeled with digoxigenin (DIG) using a RNA labeling kit (Roche Life Science, IN, USA), and the hybridized DIG-labeled RNA probes were detected by using an enzyme-linked immunoassay with a specific anti-DIG-alkaline phosphatase antibody conjugate (Roche Life Science) and a blue alkaline phosphatase chromogen (BCIP/NBT) substrate (Vector Laboratories). The blue color indicated positive signals. Sections were counterstained with nuclear fast red.

IHC was carried out, as we previously described [21]. The following primary antibodies were purchased from Santa Cruz Biotechnology: mouse monoclonal anti-AMEL antibody (1:500), rabbit polyclonal anti-AMBN antibody (1:500), goat polyclonal anti-BMP2 antibody (1:100), goat polyclonal anti-BMP4 antibody (1:100), goat polyclonal anti-BMP7 antibody (1:100), anti-P-Smad1/5/8 (1:100), Rabbit polyclonal anti-P21 antibody (Abcam; 1:400), rabbit polyclonal anti-MSX2 antibody (Abcam; 1:200) were purchased from Abcam. All the IHC experiments were performed using a DAB kit (Vector Laboratories; Burlingame, CA).

Each experiment was biologically repeated for three times.

### Western immunoblotting

Western immunoblotting was performed, as we previously described [23]. Briefly, total proteins were extracted from the enamel organ tissues of the mandibular incisors of 3-day-old *Fam20C*^*fl/fl*^ and *Sox2-Cre;Fam20C*^*fl/fl*^ mice. Western immunoblotting was then performed to detect BMP4, P-Smad1/5/8 and P21 using the corresponding antibodies described above. The secondary antibodies used were horseradish peroxidase (HRP)-conjugated goat anti-rabbit IgG (Santa Cruz Biotechnology; 1:1000) or HRP-conjugated goat anti-mouse IgG (Santa Cruz Biotechnology; 1:1000). β-actin was immunoblotted with mouse monoclonal anti-β-actin-peroxidase antibody (Sigma; 1:20,000). The immunostained protein bands were detected with ECL™ Chemiluminescent Detection reagents (Amersham Biosciences, Illinois, USA) and imaged using a CL-XPosure film (Pierce Biotechnology, Inc., New Jersey, USA).

### Statistical analyses

All quantitative data were analyzed by SPSS 13.0 software and presented as mean ± SD from 3 to 6 biological independent experiments. All data were tested for and confirmed to present normal distribution using Shapiro-Wilk test. The Student’s t-test was used to compare the means between two groups. A value of *p* < 0.05 was considered statistically significant.

## Data Availability

All data generated or analyzed during this study are included in this published article.

## Abbreviations

AI: Amelogenesis imperfecta
AMEL: Amelogenin
AMBN: Ameloblastin
ENAM: Enamelin
P21: Cyclin-dependent kinase inhibitor
Msx2: Muscle segment homeobox genes 2
EMPs: Enamel matrix proteins
MMP20: Matrix metallopeptidases 20
KLK4: Kallikrein-4
FAM20: Family with sequence similarity 20
SxE/pS: Ser-x-Glu / phospho-Ser
μCT: Micro-computed Tomography
H&E: Hematoxylin and Eosin
ISH: In situ hybridization
IHC: Immunohistochemistry
DIG: Digoxigenin
HRP: Horseradish peroxidase
